# Study of CMOS-SOI Integrated Temperature Sensing Circuits for On-Chip Temperature Monitoring

**DOI:** 10.3390/s18051629

**Published:** 2018-05-19

**Authors:** Maria Malits, Igor Brouk, Yael Nemirovsky

**Affiliations:** Department of Electrical Engineering, Technion—Israel Institute of Technology, Haifa 3200003, Israel; bigor@tx.technion.ac.il (I.B.); nemirov@ee.technion.ac.il (Y.N.)

**Keywords:** CMOS-SOI, proportional to absolute temperature (PTAT), temperature sensor, *V_t_* extractor circuit

## Abstract

This paper investigates the concepts, performance and limitations of temperature sensing circuits realized in complementary metal-oxide-semiconductor (CMOS) silicon on insulator (SOI) technology. It is shown that the MOSFET threshold voltage (*V_t_*) can be used to accurately measure the chip local temperature by using a *V_t_* extractor circuit. Furthermore, the circuit’s performance is compared to standard circuits used to generate an accurate output current or voltage proportional to the absolute temperature, i.e., proportional-to-absolute temperature (PTAT), in terms of linearity, sensitivity, power consumption, speed, accuracy and calibration needs. It is shown that the *V_t_* extractor circuit is a better solution to determine the temperature of low power, analog and mixed-signal designs due to its accuracy, low power consumption and no need for calibration. The circuit has been designed using 1 µm partially depleted (PD) CMOS-SOI technology, and demonstrates a measurement inaccuracy of ±1.5 K across 300 K–500 K temperature range while consuming only 30 µW during operation.

## 1. Introduction

Smart integrated temperature sensors and circuits are the main building blocks in all analog and mixed-signal applications, as well as in high-performance systems-on-chip (SoCs). Since the design complexity and density of VLSI circuits are increasing day by day continuous thermal monitoring is necessary to reduce thermal damage, increase reliability and avoid thermal runaway scenarios that can cause irreversible damage, i.e., overheating. The temperature across the chip should be monitored continuously, and the system operation should be adjusted accordingly. For example, in multicore SoCs, temperature information is being leveraged to maximize performance. The workload is shuffled between different cores before the temperature rises to a dangerous level. Alternatively, the clock rate is dynamically adjusted to boost system performance within a certain thermal budget [1,2,3].

To implement a comprehensive thermal monitoring system multiple temperature sensors should be used. The desired number of sensors, their exact location, and accuracy depend greatly on system-level requirements, integrated circuits (IC) packaging, and cooling system (if any). Sensor accuracy and operation range are two key system-level considerations. Typically, a less accurate sensor consumes less silicon area and power compared to a highly accurate sensor. A narrower temperature detection dynamic range relaxes the linearity requirements, which further saves area and power. It is challenging to generalize this problem, but it is widely known that the design tradeoffs are largely driven by exact application and system-level architecture. Therefore, the temperature sensors, which are used for temperature monitoring in VLSI chips should meet the following requirements: compatibility with the target process, a reasonable silicon area, low power consumption, require no additional fabrication (CMOS-SOI compatibility), low cost, high accuracy and sensor linearity in the desired temperature range.

Following these requirements, various on-chip temperature-sensing circuit have been reported in the literature. In CMOS technology, the most widely used approach takes advantage of the proportional-to-absolute temperature (PTAT) property of the voltage difference between two forward-biased diodes or bipolar junction transistors (BJT) with different currents or areas [4,5]. The accuracy of BJT-based sensors depends on the diode ideality factor and accuracy of the current ratio used to bias them. Diode ideality factor is a process-dependent parameter. Careful and meticulous layout for good matching alone cannot guarantee the required matching between the individual current mirrors to achieve the desired level of accuracy. Therefore, on-chip dynamic element matching, trimming, and post fabrication calibration techniques are used in this type of sensor to improve accuracy. As a result, thermal diodes that enable accurate temperature measurement are large and power consuming, renders them suboptimal for the whole chip thermal profiling applications where placement of a large number of sensors is necessary.

For temperature sensors operating above 500 K or uncooled thermal sensors [6] CMOS-SOI process is usually adopted due to its reduced leakage currents compared to bulk CMOS, low power consumption and availability of commercial process. Most of the CMOS based temperature sensors are not compatible with CMOS-SOI technology due to the thin device layer. The two most commonly used elements for temperature sensing available in CMOS-SOI technology are: lateral diodes [7,8,9,10,11,12] and standard MOSFET transistors [13]. The SOI-based diode is an attractive choice for a temperature sensor because it is compact in size, gives linear response up to ultra-high temperature [11] and is simpler to integrate with on-chip, sensor drive and readout circuitry. While SOI diode temperature sensors have been utilize for temperature monitoring in different application [7,8,9,10,11,12] the effect of device mismatches and consequently calibration needs are rarely discussed. One of these [8], describes a work that uses a SOI lateral PIN diode for temperature sensing from 100 K to 400 K and suggests to improve the diodes accuracy by reducing the temperature range of the diode. In other study [12], a smart CMOS-SOI temperature sensor has been described that uses a self-discharging SOI diode for temperature sensing and an error of ±1.95C (3ϭ) has been achieved after two-point calibration.

In this paper, we address the aforementioned challenges by demonstrating the design and successful implementation of a small, low-power, and accurate on chip temperature sensing circuit based on “Threshold Voltage Thermometry” [14], namely a threshold voltage (*V_t_*) extractor circuit. The performance of the *V_t_* extractor circuit is compared to a standard circuit used for temperature sensing, i.e., PTAT circuit, in terms of sensitivity, linearity, speed, accuracy, calibration needs, area and sensor power consumption. Both circuits were designed using 1 µm PD CMOS-SOI technology [15] and their performance has been experimentally verified by comparing simulated and measured results.

For 1 µm CMOS-SOI process, this 80 × 100 (µm^2^) sensor consumes ~30 μW using a 5-V supply. Our thermal measurements using multiple chips show only ±1.5 K inaccuracy between the 300 K and 500 K temperature range.

## 2. Proportional to Absolute Temperature (PTAT) Circuit

**Figure 1:**
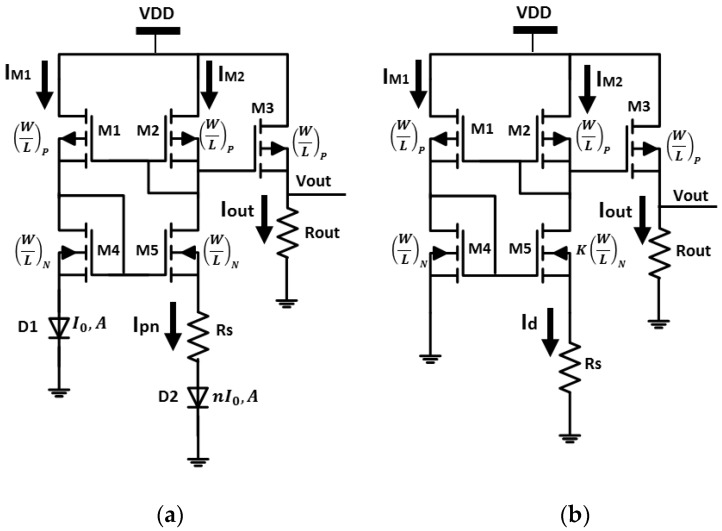
Conventional proportional to absolute temperature (PTAT) circuit implemented in CMOS technology based on (**a**) the exponential dependence of vertical PN diodes; (**b**) Polysilicon resistors.

Proportional to absolute temperature circuits are widely used to generate temperature independent current/voltage sources, band-gap reference circuits and temperature sensors in many digital, analog and mix-signal systems. Typical CMOS based PTAT circuits are shown in Figure 1a,b [16]. The first circuit is based on the exponential dependence of the vertical PN diode forward voltage upon temperature and the second one is based on the dependence of resistors and channel mobility to generate an output voltage/current proportional to the chip local temperature.

### 2.1. Principle of Operation

The sensing principle of a PTAT temperature sensor is depicted in Figure 1a. The sensor’s core consists of a pair of matched vertical PN diodes biased by two identical current sources (*I_pn_*) while diode D2 consists of n parallel connected diodes with the same device area [7]. The voltage drop on each diode is given by [14]:

(1) $$V_{pn}=\;\frac{k_{B}T}{q}[\ln\;(\frac{I_{pn}}{I_{0}}+1)]\approx \frac{k_{B}T}{q}\ln\;(\frac{I_{pn}}{I_{0}})$$

where *I_pn_* is the diode current, *q* is the electron charge, *k_B_* is Boltzmann’s constant, *V_pn_* is the voltage across the diode, *T* is the absolute temperature, *I*_0_ is the temperature-dependent reverse saturation current.

By choosing the upper transistors so that the current in both branches is similar (*I_M_*_1_ = *I_M_*_1_ = *I_pn_*), and by neglecting channel length modulation and bulk effect the circuit output voltage can be calculated:

(2) $$\begin{array}{l} V_{pn,D1}=V_{pn,D2}+I_{pn}\cdot R_{s}\\ \frac{k_{B}T}{q}\ln\;(\frac{I_{pn}}{I_{0}})\;=\frac{k_{B}T}{q}\ln\;(\frac{I_{pn}}{nI_{0}})+I_{pn}\cdot R_{s}\\ I_{pn}=I_{out}=\frac{k_{B}T\ln(n)}{qR_{s}}\\ V_{out}=I_{out}\cdot R_{out}=\frac{k_{B}T}{q}\cdot \ln(n)\cdot \frac{R_{out}}{R_{s}} \end{array}$$

where *n* is the number of parallel connected diodes, *I_out_* is the circuit output current, *R_out_* and *R_s_* are the output and series resistors respectively. Usually, the resistors *R_out_* and *R_s_* are from the same type so their temperature coefficient will balance each other and improve the circuit performance.

Figure 1b illustrates another PTAT implementation which uses the dependence of mobility and resistors upon temperature to generate the output voltage. The upper *p*-type transistors (M1 and M2) have the same current (*I*_M1_ = *I*_M2_ = *I_d_*) because they have identical dimensions. Hence, we can calculate the NMOS gate voltage:

(3) $$V_{gs4}=V_{g5}+I_{d}\times R_{s}$$

where *V_gs_* is the transistor gate-source voltage and *I_d_* is the transistor drain current.

By neglecting body effect we can determine the circuit output voltage:

(4) $$\begin{array}{l} \sqrt{\frac{2I_{d}}{\mu_{n}C_{ox}{\left(W/L\right)}_{N}}}+V_{t4}=\sqrt{\frac{2I_{d}}{\mu_{n}C_{ox}K{\left(W/L\right)}_{N}}}+V_{t5}+I_{d}\times R_{s}\\ \sqrt{\frac{2I_{d}}{\mu_{n}C_{ox}{\left(W/L\right)}_{N}}}\left(1-\frac{1}{\sqrt{K}}\right)=I_{d}\times R_{s}\\ I_{d}=I_{out}=\frac{2}{\mu_{n}C_{ox}{\left(W/L\right)}_{N}}\times \frac{1}{R_{s}^{2}}{\left(1-\frac{1}{\sqrt{K}}\right)}^{2}\\ V_{out}=I_{out}\times R_{out}=\frac{2}{\mu_{n}C_{ox}{\left(W/L\right)}_{N}}\times \frac{R_{out}}{R_{s}^{2}}{\left(1-\frac{1}{\sqrt{K}}\right)}^{2} \end{array}$$

where *µ_n_* is the electron mobility, *K* is transistors M4 and M5 size ratio, *W* and *L* are the transistor width and length respectively and *C_ox_* is the oxide capacitance.

As seen from Equation (4), the output voltage is inversely proportional to the channel mobility creating a proportional to absolute temperature output voltage. It is important to emphasize that since each diode-connected device feeds from a current source this design is relatively independent of *V_dd_*.

### 2.2. Implementation

**Figure 2:**
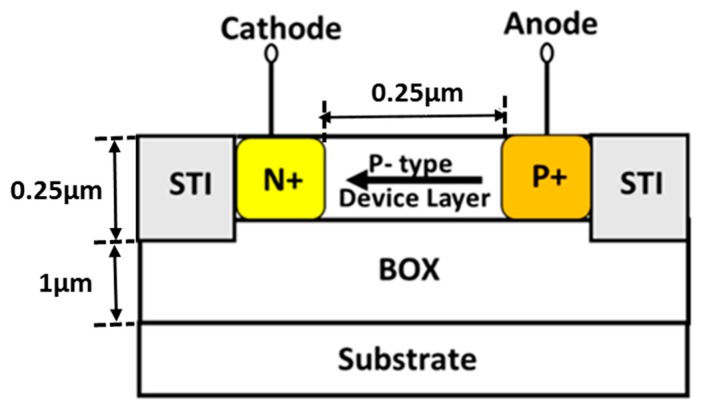
Schematic cross-section of the lateral SOI diode used in the diode based PTAT circuit.

In CMOS-SOI technology it is impossible to manufacture a vertical diode due to the thin body layer; hence, in order to implement a diode based circuit (Figure 1a), a forward-biased diode is built with a lateral structure based on the device layer, forming a source/drain PIN junction. The diode based circuit was implemented using 25 N+/P-well/P+ non-gated diodes (24 are finger diodes interdigitated in parallel) with width of 16 µm and length of 0.25 µm, as presented in Figure 2. The PMOS and NMOS dimensions are (*W*/*L*)*_P_* = 6 µm/2 µm/(*W*/*L*)*_N_* = 6 µm/3 µm for the diode based design and (*W*/*L*)*_P_* = 24 µm/2 µm/(*W*/*L*)*_N_* = 6 µm/3 µm for the resistor based design. The size ratio between transistors M4 and M5, i.e., *K*, in the resistor based PTAT (Figure 1b) is 2.

Both architectures are realized in a standard 1 µm PD CMOS-SOI process [15]. The maximum operating voltage is 5 V. The buried oxide (BOX) thickness is 1 μm, the gate oxide thickness is 25 nm, and the active silicon thickness is 250 nm. The circuits chip areas are 178 × 150 µm^2^ and 85 × 100 µm^2^ for the diode based design and the resistor based design, respectively. The resistors in both circuits are implemented using high resistivity Polysilicon.

Although the SOI lateral diode is modeled as an ideal diode, it should be noted that the saturation current exhibits perimeter dependence rather than area dependence, as in regular planar bulk diodes, due to the thin body layer. Contrary to CMOS bulk diodes, where surface effects may be neglected, the thin device layer in SOI technology requires a model where the current is primarily dependent on surface effects, i.e., *I*_0_ = *J_SW_*(*T*) × Perimeter.

As a result, the saturation current is strongly affected by the surface, determined by the device periphery. This significantly increases the mismatch between diodes and affects the diode’s performance as a temperature sensor, as reported in [14] for lateral diodes fabricated in two different SOI processes.

### 2.3. Measurements and Simulations

**Figure 3:**
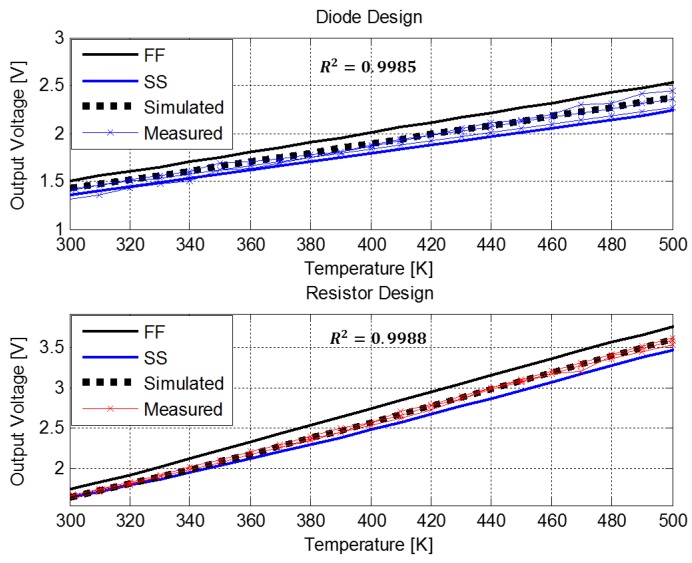
Measured and simulated output voltage of three chips for both PTAT designs at a temperature range of 300 K–500 K. Dots: experimental data; dashed lines: simulation results using the SPICE circuit simulator based on and BSIM4 MOSFET models; solid: circuit output voltage in different process corners (FF and SS).

The output voltage of both designs is sampled by using a DMM4040 Digital Precision Multimeter (Tektronix, Beaverton, OR, USA) while the circuit’s temperature is determined with a variable temperature micro probe system from MMR Technologies (San Jose, CA, USA), which features a temperature control accuracy of ±0.01 K. All transistors (M1–M5) are biased and their current measured using a B1500A semiconductor parameter analyzer (Agilent, Santa Clara, California, USA). Three samples from each design were characterized in temperature ranging from 300 K to 500 K and the measured output voltage is shown in Figure 3. These results are compared to *V_out_* vs. *T* curves obtained from electrical simulations in the SPICE simulator based on BSIM4 MOSFET models [17], also presented in Figure 3.

The temperature sensing circuits’ performance is also analyzed using process corner simulations. A four-corner model file provided by the foundry is used for the corner-based analysis. In Figure 3 only the corners with worst case variations are shown—Fast NMOS Fast PMOS (FF) and Slow NMOS Slow PMOS (SS).

In this analysis, the coefficient of determination, R^2^ [18] has been used to evaluate the linearity of the sensor, through the agreement between the experimental data and their best linear fit. Figure 3 shows that the resistor based design exhibits linear dependence upon temperature in the entire temperature range (R^2^ = 0.9988 in the worst case sample) and minimal variations between the measured samples. The diode based design is also linear as a function of temperature (R^2^ = 0.9985 in the worst case sample), but has larger variations between the different samples. The measured temperature sensitivity of the output voltage after a linear curve fit is 9.8 mV/K for the resistor based PTAT design and it is close to that obtained from the simulation. The sensitivity of the diode based design is 4.7 mV/K for a bias current of 3.3 µA, which is higher than that reported for SOI lateral PIN diodes, which is in the order of 1.1 mV/K for a bias current of 2 µA [7,8,9].

As illustrated in Figure 3, for the diode based design there is a DC shift between the measured and simulated results for all measured samples (maximum value of DC shift is 32 mV) due to the dependence of the saturation current in the diode dimensions. This dependency of the saturation current causes a large mismatch between lateral diodes that should be identical, as reported by us in [14]. As a result, the accuracy of the diode based PTAT circuit decreases.

In addition, the corner analysis shows that the worst case maximum temperature error occurs at the FF corner and caused combined offset of 100 mV and 150 mV for the resistor and diode based PTAT designs. It is important to note that this offset includes large mismatches in polysilicon resistance value and can be improved by matched layout. The shifts and variations between the different measured samples for the diode based design results in large temperature measurement errors and require precise calibration process.

**Figure 4:**
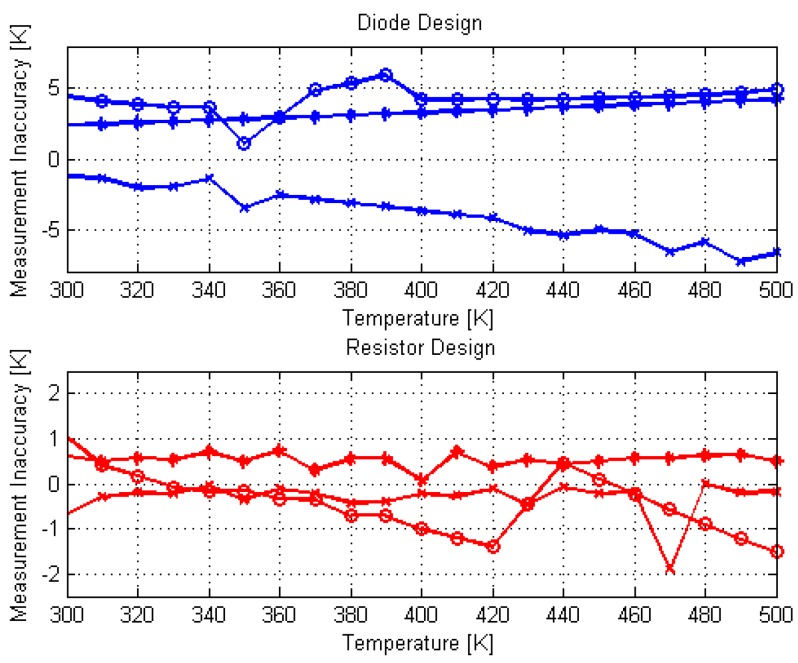
Measurement inaccuracy versus temperature for three different chips in both PTAT designs.

The error in the temperature measurement (Δ*T*) has been obtained by calculating the difference, Δ*V_out_*, between the measured and simulated *V_out_* vs. *T*, which is then converted into temperature using the circuit sensitivity. Figure 4 shows the error in temperature measurement as a function of applied temperature for both designs in the measured samples presented in Figure 3. Accordingly, the sensor’s inaccuracy is estimated:

(5) $$\Delta T=\frac{\left|V_{out,measured}-V_{out,simulated}\right|}{dV_{out}/dT}$$

From the extracted error curves presented in Figure 4 one can observe a maximum error of ±6.5 K for the diode based PTAT circuit and an error of ±1.5 K when using the temperature dependence of polysilicon resistors, without any additional calibration. These results show that the resistor based PTAT circuit is a good temperature sensor for temperatures ranging from 300 K till 500 K, though it is power consuming. The circuit measured power consumption at room temperature is 0.25 mW for the resistor based design and 45 µW for the diode based design. Errors similar to the errors calculated for the resistor based design, in order of 2 K, have been presented in [12] after two-point calibration for temperature sensing from 278 K up to 373 K using fully depleted single lateral SOI diode consuming 100 µW.

### 2.4. Time Response

In addition to the steady-state on-chip temperature distribution, the sensor’s thermal transient response can also be of interest for various applications. For instance, in dynamic thermal/leakage management, the dynamic variation of on-chip temperature is used to adjust the operation of the chip such that the leakage power and the peak chip temperature can be properly controlled [19].

**Figure 5:**
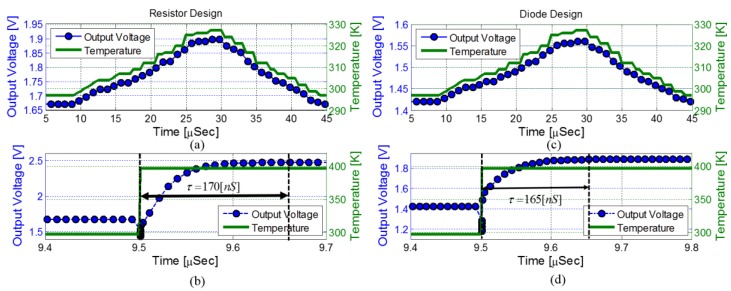
Simulated time response to a graduate local temperature change (**a**,**c**) and to a step change in local temperature (**b**,**d**) for both PTAT designs.

The time response of a temperature sensor is defined as the time it takes the sensor output to achieve 63% of its final value after a step change in temperature is impressed on its surface. To perform thermal transient analysis, a numerical integration method such as the backward Euler is required [20]. To solve the thermal transient analysis problem, one can model the thermal system as an equivalent RC circuit. Then, a SPICE-like simulation technique can be applied to the equivalent RC circuit to provide the thermal transient response [20,21]. In this study the PTAT time response was simulated using SPICE circuit simulator with BSIM4 MOSFET models [17]. The standard MOSFET models were changed to include a parameter describing each device specific temperature rise above the simulated ambient temperature, i.e., *t_rise_*. Then, in order to simulate a local temperature, only trise of the MOSFETs composing the temperature sensing circuit were changed externally and the PTAT output voltage was sampled. The transient simulation results are presented in Figure 5 for both PTAT designs and two temperature profiles: a graduate and a step temperature change.

Figure 5a,c show the PTAT response to a graduate temperature change; from *t* = 10 µs to *t* = 30 µs the circuit local temperature increases from 295 K to 330 K (*t_rise_* = 35 K) and then it starts to cool down to room temperature. This is a lifelike scenario for most CMOS-SOI applications were the thermal time constants are long [22]. It can be easily concluded that both PTAT designs follow the temperature changes accurately and without delay, meaning that the time response of the circuit is much smaller than chip thermal time constants.

The time response of each design is obtained by simulating a step change in the sensor local temperature and the results are presented in Figure 5b,d. Time constants of 170 ns and 165 ns were calculated for the resistor and diode based designs, respectively.

## 3. *V_t_* Extractor Circuit

We previously proposed to determine the chip local temperature by measuring the transistor threshold voltage [14]. This requires a careful thermal characterization of *V_t_*(*T*) and d*V_t_*/d*T* of the process under study. Subsequently, by monitoring the changes in *V_t_* under actual operation, the true local temperature of devices can be determined. We refer to this method as “Threshold—Voltage Thermometry” and we recently described it in details in [14]. In order to implement this method the MOSFET threshold voltage needs to be extracted during the chip operation, i.e., on-line. This was done by using a *V_t_* extractor circuit [23,24,25,26,27]. A *V_t_* extractor is a circuit that extracts the threshold voltage of a MOS device according to the device local temperature [23].

### 3.1. Principle of Operation

The architecture for a *V_t_* extractor circuit which we have chosen to implement is based on [28]. We chose this design because it combines a simple low voltage *V_t_* extracting block and feedback, to achieve independence of the output from the supply voltage, low current consumption (therefore, low consumption), accuracy of the extracted threshold voltage toward supply voltage variations and transistor mismatch.

**Figure 6:**
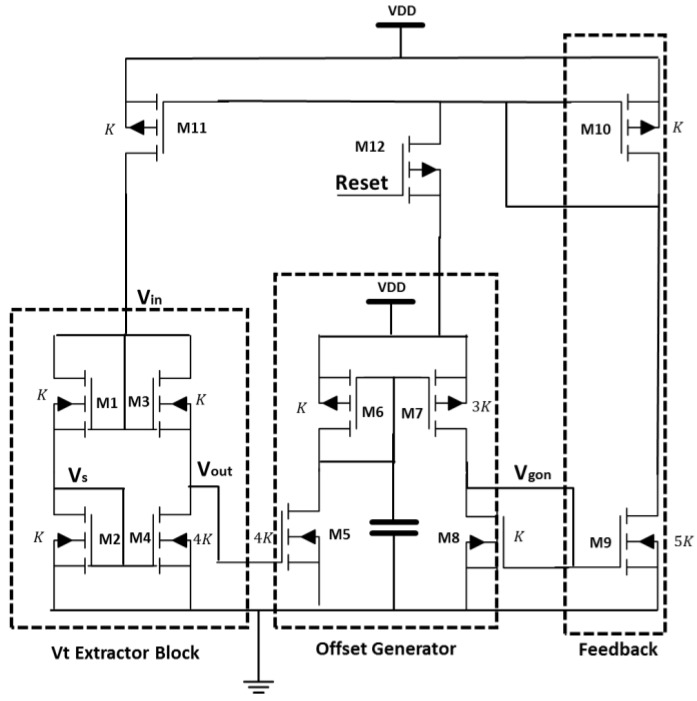
Schematic of *V_t_* Extractor circuit.

The tested extractor is presented in Figure 6 and consists of three blocks: (i) a simple *V_t_* extracting block; (ii) an offset generator; and (iii) the current feedback loop.

#### 3.1.1. Analysis of *V_t_* Extracting Block

Assuming transistors M1 till M4 are all operating in saturation, with the ratios of (*W*/*L*) shown in Figure 6 ($K_{1}=K_{2}=K_{3}=K_{4}/4=K$), eliminating body effect ( *V_bs_*_,*i*_ = 0) by connecting the bulk and source terminals in order to improve the circuit accuracy, and assuming that the drain current (*I_d_*) of each MOSFET follows the simple quadratic law:

(6) $$I_{d_{i}}=K_{i}{\left(V_{gs_{i}}-V_{t}\right)}^{2}$$

then:

(7) $$\begin{array}{l} I_{d1}=I_{d2}\Rightarrow V_{s}=V_{in}/2\\ I_{d3}=I_{d4}\Rightarrow V_{out}=V_{t} \end{array}$$

where the voltage nodes (*V_s_*, *V_in_* and *V_out_*) are shown in Figure 6 and *K_i_* is the transcoductance of transistor *i*.

In order to compensate for channel length modulation, mobility reduction and transistor mismatch the following feedback to the *V_t_* extractor block (*V_in_*) is needed [23]:

(8) $$V_{in}=2V_{out}+2V_{off}$$

where *V_off_* is several (*k_B_T/q*).

#### 3.1.2. Analysis of Offset Generator

In the attempt to implement the feedback shown in Equation (8), an offset should be added to *V_out_*. The purpose of the offset generator is to take the *V_t_* extractor block output and add an offset of several (*k_B_T/q*) to this value. Considering that *V_out_* is close to *V_t_*, then       *V_gs5_ – V_t_ < 3k_B_T/q*, subthreshold drain current equations are used in order to calculate this block’s output −*V_gon_*:

(9) $$\begin{array}{l} aI_{d5}=I_{d8}\Rightarrow aI_{0}\exp(V_{out}/(k_{B}T/q))=\frac{I_{0}}{b}\exp(V_{gon}/(k_{B}T/q))\\ \Rightarrow V_{gon}=V_{out}+(k_{B}T/q)\ln(ab) \end{array}$$

where *a = K*_7_/*K*_6_ is the p-mirror current gain and b is the ratio *K*_5_/*K*_8_. Hence, *V_gon_* includes the necessary offset from *V_out_*.

#### 3.1.3. Analysis of Feedback Block

Transistors M9 and M4 in Figure 6 are 5 and 4 times wider than M2, respectively, and due to the current mirror formed by M10 and M11, *V_gon_* is fed back to *V_s_* as follows:

(10) $$\begin{array}{l} I_{d9}=I_{d2}+I_{d4}\Rightarrow V_{s}=V_{gon}\overset{\left(6)+(9\right)}{\Rightarrow }\\ V_{in}=2V_{out}+2(k_{B}T/q)\ln(ab) \end{array}$$

so the proposed feedback implements Equation (10), corresponding to Equation (8) resulting in a very accurate extraction of the threshold voltage.

### 3.2. Implementation

The circuit was implemented in 1 µm PD CMOS-SOI process [15]. The MOSFET (*W*/*L*) ratio was chosen by performing a large number of parametric simulations in which the circuit output was compared with the simulated threshold voltage calculated using BSIM4 MOSFET [17] models at different temperatures. For supply voltage of 5 V, an optimal performance in terms of accuracy, chip area and circuit power consumption was achieved for body connected transistors with body contact shortened to the source ($V_{bs_{i}}=0$) and *W*/*L* ratio of 4/9 µm/µm for all transistors in the *V_t_* extractor, offset generator and feedback blocks. The capacitor in the offset generator block is used to prevent parasitic oscillation and noise disturbances during transient. The resent transistor (M12) is set with minimum sizing (*W*/*L* = 4/1 µm/µm) and used as an on/off switch for the *V_t_* extractor circuit. 

Overall, the circuit occupies a chip area of 80 × 100 µm^2^ and its measured power consumption at room temperature is 27.5 µW.

### 3.3. Measurements and Simulations

The *V_t_* extractor voltage was measured at several nodes, see Figure 6, in temperatures ranging from 300 K to 500 K. The measured output voltage is compared to circuit DC simulations, the nominal threshold voltage obtained from BSIM4 MOSFET [17] models and process thermal characterization in order to determine the sensors accuracy. In addition, process corner simulations were run to predict linearity robustness over process variations compared to typical operation.

**Figure 7:**
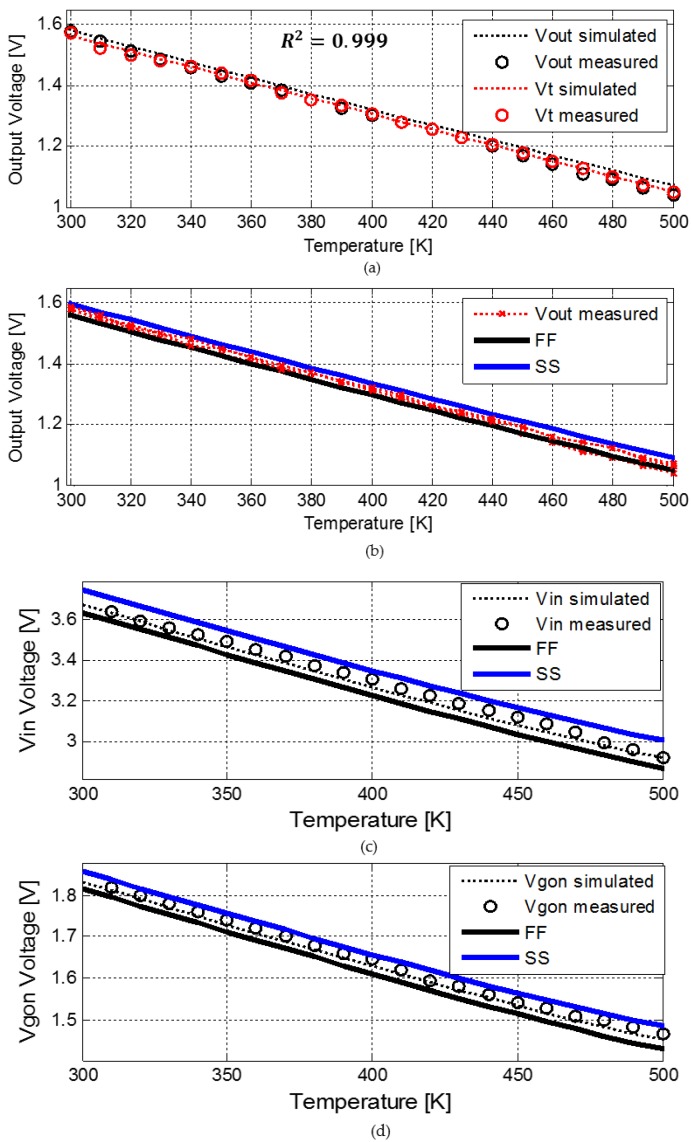
Comparison of the measured and simulate *V_t_* extractor circuit different voltages (**a**) Vout and *V_t_* (**b**) measured *V_out_* for three different chips (**c**) *V_in_* and (**d**) *V_gon_*. Dots: experimental data; dashed lines: simulation results using the SPICE circuit simulator based on and BSIM4 MOSFET models; solid: circuit voltage in different process corners (FF and SS).

Figure 7 shows the measured and simulated temperature dependence of the *V_t_* extractor circuit voltages (*V_out_, V_in_ and V_gon_*) for three different samples from 300 K to 500 K. The threshold voltage calculated using BSIM4 MOSFET [17] model and the one extracted during the process thermal characterization are presented as well. In Figure 7 only the corners with worst case variations are shown—FF and SS.

To evaluate the linearity of the *V_t_* extractor circuit the coefficient of determination R^2^ [18] has been used like in the PTAT sensors case. As seen from Figure 7a the circuit output is linear with temperature in the entire temperature range (R^2^ = 0.999 for all measured samples) and produces a maximum error of ~1% (~12 mV DC shift) from the simulated and measured *V_t_* values for all measured samples. At high temperatures, the increase in the excess voltage (*V_off_*) can cause nonlinearity in the temperature sensing capability of the *V_t_* extractor circuit due to the offset created between the desired feedback, as indicated in Equation (8), and *V_in_*. Hence, if the generated *V_in_* is too high, the transistors in the *V_t_* extractor block (presented in Figure 6) won’t operate in saturation causing nonlinearity of the output voltage.

The measured sensor sensitivity given by the slope of *V_out_* vs. *T* shown in Figure 7a is 2.6 mV/K, which is close to the threshold voltage dependence upon temperature (d*V_t_*/d*T*) obtained from simulation (−2.5 mV/K) and is consistent over the three samples. There is a good correspondence between all measured and simulated results for all sampled circuit voltages (*V_out_*, *V_in_* and *V_gon_*), as shown in Figure 7a–d. The corner analysis demonstrates that the maximum temperature error due to process variations occurs for the SS corner and caused a maximum offset of 25 mV in the circuit output voltage. In addition, as seen in Figure 7b, there are minor variations between the different measured samples which verifies the robustness, independence of the output voltage upon transistor mismatch and repeatability of this design.

The measurement inaccuracy in the temperature sensing (Δ*T*) has been obtained by calculating the difference, Δ*V*, between the circuits measured output voltage and the threshold voltage extracted during the process thermal characterization, which is then converted into temperature using the measured d*V_t_*/d*T*. Accordingly, the error in temperature is estimated by:

(11) $$\Delta T=\frac{\left|V_{out,measured}-V_{t,measured}\right|}{dV_{t}/dT}$$

**Figure 8:**
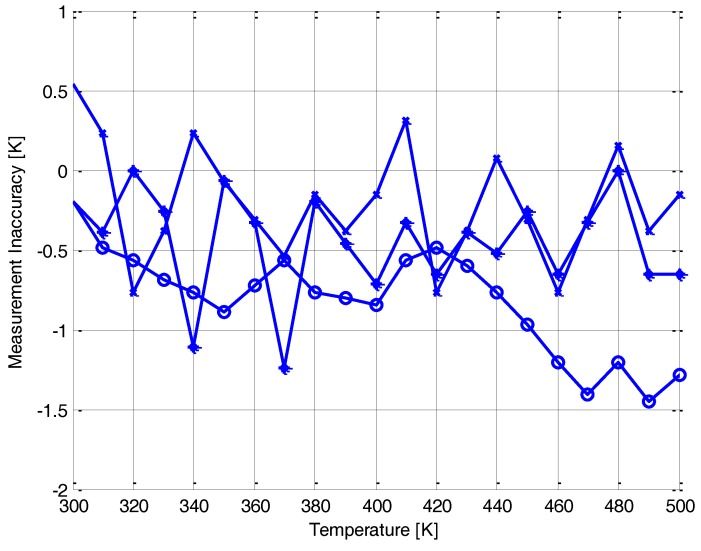
Measurement inaccuracy versus temperature for three different chips.

Figure 8 shows the temperature error as a function of applied temperature for three different chips. The worst-case errors occur at high temperatures (~500 K) most likely due to the excess voltage *V_off_*, which is proportional to the thermal voltage (*k_B_T/q*), causing a large variation from the desired feedback as indicated in Equation (8). After applying common-centroid and other matching techniques in the layout the maximum error due to offset is 1.5 K around 500 K, as shown in Figure 8. At the low end of the temperature range near room temperature, the error is much less, around 0.5 K. In future design, offset cancellation techniques such as chopping and auto-zeroing can be applied to further reduce the effect of offset on the sensor accuracy and improve the performance.

### 3.4. Time Response

**Figure 9:**
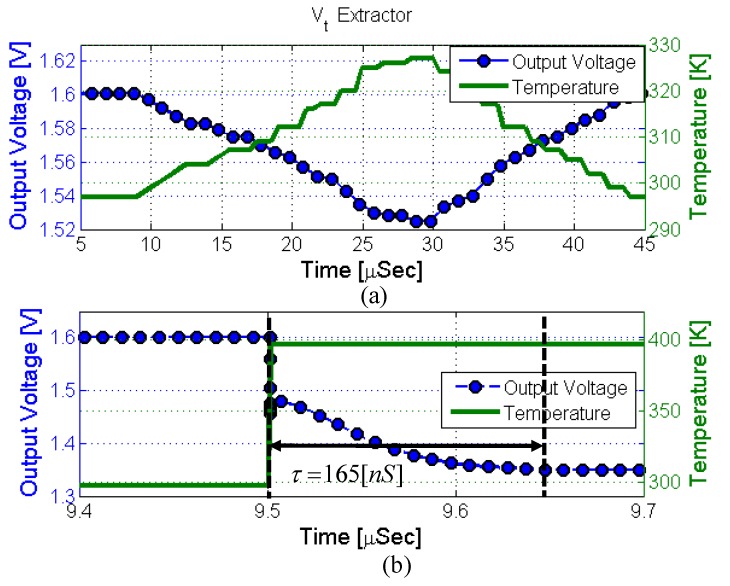
Simulated time response to a (**a**) graduate local temperature change and to a (**b**) step change in local temperature.

The time response of the *V_t_* extractor circuit was simulated under the same conditions (graduate and step change in temperature) as the PTAT and the results are shown in Figure 9. Figure 9a present the *V_t_* extractor response to a graduate temperature change and it can be easily seen that the sensor follows the temperature changes accurately and without delay; meaning a decrease in the output voltage when temperature increases and vice versa during the chip cool down.

The time response of the sensor is obtained by simulating a step change in the sensor local temperature and the results are presented in Figure 9b. A Time constant of 165 ns was calculated from this simulation.

## 4. Summary

In this paper the thermal performance of various circuits for temperature sensing and monitoring manufactured in 1 µm PD CMOS-SOI technology was characterized and compared in the temperature range of 300 K–500 K. All sensors exhibit linearity and high sensitivity over the entire temperature range. However, the lateral diodes based PTAT design is inaccurate due to diode mismatches caused by the dependence of the saturation current upon diode dimensions. A resistor based PTAT has good accuracy but requires high power consumption during operation. The PTAT circuit is best utilized when implementing temperature independent current or voltage sources (Band Gap References), although a carful calibration is needed. The implemented *V_t_* extractor circuit has a small error (1%) under nominal conditions, linear dependence upon temperature, low power consumption, low area and fast response time. This makes this sensor optimal to be used as a temperature sensor for thermal management in CMOS-SOI technologies. A performance comparison between the sensors reported in this paper and the recent on-chip temperature sensors is shown in Table 1. As evident, the *V_t_* extractor circuit presented here is smaller than most reported sensors and has low power consumption while achieving good accuracy with no need in additional calibration. The circuit will be useful as a temperature sensor in high-performance analog, mixed-signal, and digital ICs due to its high performance and low power consumption. Other applications for this temperature sensor are IR sources or detectors and new generation of smart sensors, like gas sensors, where temperature monitoring is necessary to achieve better sensitivity and selectivity in presence of different gases [6].

## Figures and Tables

**Table 1:** Performance comparison.

Sensor	Process	Range (K)	Area (mm^2^)	Power (µW)	Maximum Sensitivity (mV/K)	Linearity (R^2^)	Accuracy (K)	Calibration Need
[7]	150 nm CMOS SOI	150–400	-	-	0.7	0.99	-	Needed
[9]	FD CMOS SOI	100–400	0.04	-	−2.2	-	2	Needed
[10]	1 µm CMOS SOI	300–1053	-	27	−1.22	-	-	-
[12]	32 nm CMOS SOI	270–370	0.001	100	-	-	1.95 (3ϭ)	Needed
[13]	1 µm PD CMOS SOI	300–500	0.45	112.5	-	-	2	Needed
[24]	0.35 µm CMOS	250–350	0.055	0.3	15 (ppm/C)	-	-	-
[25]	1 µm PD CMOS SOI	250–520	0.23	-	27 (ppm/C)	-	1.8 (%)	Needed
[27]	0.35 µm CMOS	270–400	0.011	24	11.8 (ppm/C)	-	0.153 (%)	-
Resistor based PTAT(This work)	1 µm PD CMOS SOI	300–500	0.0085	250	9.8	0.9988	1.5	Needed
Diode based PTAT(This work)	1 µm PD CMOS SOI	300–500	0.026	45	4.7	0.9985	6.5	Needed
V_t_ Extractor Circuit(This work)	1 µm PD CMOS SOI	300–500	0.008	27.5	2.6	0.999	1.5	Not Needed
